# The Association of Positive or Negative Religious Coping Methods With Psychological Distress and Quality of Life Among Parents of Infants With Congenital Heart Disease

**DOI:** 10.3389/fped.2021.753032

**Published:** 2021-11-19

**Authors:** Jian-Feng Liu, Wen-Peng Xie, Wen-Hao Lin, Hua Cao, Qiang Chen

**Affiliations:** ^1^Department of Cardiac Surgery, Fujian Branch of Shanghai Children's Medical Center, Fuzhou, China; ^2^Department of Cardiac Surgery, Fujian Children's Hospital, Fuzhou, China; ^3^Fujian Maternity and Child Health Hospital, Affiliated Hospital of Fujian Medical University, Fuzhou, China; ^4^Fujian Key Laboratory of Women and Children's Critical Diseases Research, Fujian Maternity and Child Health Hospital, Fuzhou, China

**Keywords:** religion, psychological distress, quality of life, CHD, parent

## Abstract

**Objective:** The purpose of this study was to evaluate whether positive and negative religious coping methods were associated with psychological distress and quality of life in parents of infants with congenital heart disease (CHD).

**Methods:** This descriptive, cross-sectional study was conducted at a provincial hospital in Fujian, China. Clinical data from 115 parents of infants with CHD were collected. Chinese Sociodemographic Forms, Brief RCOPE, Beck Depression Interview (BDI), and the Short Form Health Survey (SF-36) were used in this study.

**Results:** The sex of caregivers in infants with CHD was an independent predictor of BDI scores. The positive religious coping score and the negative religious coping score were both independent predictors of the BDI score (β = −5.365, *P* = 0.006 and β = 4.812, *p* = 0.017). The correlation between the quality-of-life scores and positive or negative religious coping scores indicated that positive religious coping scores were significantly positively correlated with Vitality, Social Functioning, and Mental Health scores. There was a significant negative correlation between negative religious coping scores and mental health scores.

**Conclusions:** Positive or negative religious coping methods may be associated with psychological distress and quality of life among parents of infants with CHD. It is suggested that more attention should be devoted to the influence of religious coping methods on parents of infants with CHD, and the use of religious resources should be encouraged.

## Introduction

Congenital heart disease (CHD) is one of the most common structural malformations in live births. The number of new CHD cases worldwide is estimated to be 1.35 million, and the incidence of CHD in newborns is 1%. China has more than 100,000 new CHD cases every year (1, 2). With the improvement of medical technology in recent years, the survival rate of infants with CHD has dramatically improved (3). However, these advances in medical technology have not been accompanied by an improved understanding of the psychological effects on the families of infants with CHD. Parents of infants with CHD, especially mothers, are more likely to suffer from mental health problems and psychological distress because of heart surgery, extended hospital stays, feeding difficulties, and financial and emotional stress (4–6). Additionally, these psychological problems will affect the quality of parental care for infants and their quality of life. Therefore, effective coping methods are needed to address their psychological problems so that they can concentrate more on caring for their infant and improve their quality of life.

Positive or negative religious coping methods have been reported as a coping strategy for negative life events. In a study of religious coping in Asia, religious coping was the most common method used by parents of sick infants experiencing emotional disorders (7). However, there is minimal knowledge about the impact of positive or negative religious coping methods on psychological distress among parents of infants with CHD. In addition, the relationship between positive or negative religious coping methods and the quality of life among parents of infants with CHD is unknown. The purpose of this study was to investigate the association of positive or negative religious coping methods with parental psychological distress and quality of life.

## Methods

This descriptive, cross-sectional study was conducted from February 2020 to February 2021 at a provincial hospital in the Fujian province in China. The ethics committee of our hospital approved the study protocol, and informed consent was obtained from all the parents. The inclusion criteria were as follows: (1) parents of infants who had been diagnosed with simple CHD; (2) parents were the primary caregivers; (3) selected only the primary caregiver of the parent; and (4) participants had explicit religious beliefs. The exclusion criteria were as follows: 1. caregivers were not the parents; (2) the patient was in serious condition and needed emergency surgery; (3) the patient was complicated with other congenital or systemic diseases; and (4) the patient's parents refused to participate in this study.

GPower 3.1.9.2 was used for sample size calculation. Based on the results of previous preliminary surveys, we anticipated a medium effect size. We set the alpha value to 0.05 and the statistical power to 0.90. The statistical results indicated that at least 109 parents should be included in the study. Considering a potential 10% loss of follow-up rate, we planned to recruit 120 parents. In this study, five participants were excluded from the study: three participants were not the primary caregivers, and the other two participants decided to withdraw from the study for personal reasons. In the end, the study successfully recruited and collected data from 115 parents of infants with simple CHD, all of whom spent considerable time caring for the patients. The patients' diagnostic components were ventricular septal defects (n = 56), patent ductus arteriosus (n = 31), atrial septal defects (n = 18), and pulmonary stenosis (n = 10).

### Evaluation Tool

Brief RCOPE was used to evaluate religious coping. The scale consists of fourteen items to measure the respondent's religious coping methods. The reliability and internal consistency estimation were high, with Cronbach's alpha values of 0.809 and 0.931 (8). After verification, the scale also had high validity and internal consistency in the Asian population (9). In this study, the Chinese version of the Brief RCOPE was used. The Brief RCOPE was designed to provide an efficient, theoretically meaningful way to integrate religious dimensions into models and research on stress, coping, and health. The scale consists of seven positive response items (P COPE) and seven negative response items (N COPE). Considering that the expressions of religions may vary with religious belief groups and cultures, we made appropriate modifications to some expressions of the Brief RCOPE scale to make their expressions more explicit about retaining the meaning of the original text and increased the cultural adaptability of the scale. For example, we changed from “Wondered whether my church had abandoned me” to “Wondered whether my church/temple/mosque or my religious leader had abandoned me.” The score for each item ranges from 0 (not at all) to 3 (very). The total score for positive and negative items ranges from 0 to 21.

The severity of depression was assessed by the Beck Depression Interview (BDI) (10). The Chinese version of the BDI has been widely used in clinical patients and general population research in China. The BDI is composed of 21 items. Each item is scored on a four-point scale (ranging from 0 to 3), and its total score is used to assess the severity of depression symptoms (BDI total score: 0 to 15: no depression, 16 to 20: mild depression, 21 to 29: moderate depression, score ≥30: severe depression) (11).

The Chinese version of the Short-Form Health Survey (SF-36) has been verified to have good internal consistency and reliability (12). There are 36 items on the SF-36 scale. Item 2 asks about self-reported health change and is not included in scoring. The remaining 35 items constitute physical functioning (PF), role-physical (RP), bodily pain (BP), general health (GH), vitality (VT), social functioning (SF), role emotional (RE), and mental health (MH). Each dimension was encoded, the scores of the questions were summarized, and the total score was converted to a score of 0 (the worst quality of life) to 100 (the best quality of life). Although there is no consensus on the best cutoff value to use, a review of the accuracy of a single SF-36 total score found that using 50 as a cutoff value can strike the best balance between sensitivity and specificity. Therefore, a total score of SF-36 ≤ 50 indicates that quality of life (QoL) is impaired.

### Data Collection

Two recruitment methods were used in this study: on-site and online recruitment. For on-site recruitment, the researchers invited the parents who had follow-up physical examinations in the outpatient clinic to participate in the study. Participants filled out questionnaires on-site. For online recruitment, through the WeChat platform, the parents could follow a procedure similar to the on-site investigation. The data were collected by the research assistants who were independent of this study and received special training. The research assistants explained to the participants the nature and purpose of this study, the principle of voluntary participation, and the confidentiality of the information. Parents who agreed to participate in this study signed informed consent forms and were asked to complete the Chinese version of the Social Demographic Form, Brief RCOPE, Beck Depression Interview (BDI), and Short Form Health Survey. Infant information was collected, including general clinical data about age, sex, and type of CHD.

### Statistical Analysis

Quantitative variables were expressed as the means and standard deviations (SD), while qualitative variables were expressed as frequency and percentage values (%). All statistical tests were two-tailed, and a significance level of *p* < 0.05 was used for the statistical tests. Multiple linear regression analysis was performed to compare the relationship between BDI and age, sex, and positive or negative religious coping methods. The Kolmogorov-Smirnov (K-S) test was used to assess whether the data conformed to a normal distribution. Spearman's correlation analysis was used to explore the relationship between positive or negative religious coping styles and quality of life. The data were analyzed using IBM SPSS 22 software.

## Results

Table 1 describes the sample characteristics of this study. A total of 115 parents of infants with CHD met the inclusion criteria and were successfully included in this study. The positive religious coping score was 18.9 ± 6.7, and the negative religious coping score was 11.2 ± 4.1. The parents' BDI score was 17 ± 5.9.

**Table 1:** Comparison of demographic characteristics of children and their parents between the two groups.

	***n* (%)**	**mean ± SD**
Patient demographic characteristics		
Female gender of patient	50 (43.5)	
Age, m		2.9 ± 1.2
Caregiver demographic characteristics		
Female gender of Caregiver	89 (77.4)	
Age, y		27.6 ± 13.4
Religious affiliation		
Buddhism	63 (54.8)	
Catholicism	17 (14.8)	
Muslim	10 (8.7)	
Protestant	14 (12.2)	
Other	11 (9.5)	
Brief RCOPE		
Positive coping method		18.9 ± 6.7
Negative coping method		11.2 ± 4.1
BDI score		17 ± 5.9
SF-36 score		
Physical function		71 ± 22.5
Role-physical		45 ± 31.3
Bodily pain		67 ± 17.5
General health		59 ± 26.6
Vitality		76 ± 32.8
Social functioning		55 ± 28.2
Role-emotional		38 ± 15.2
Mental health		41 ± 21.1
Parents' educational level		
Below senior high school	23 (20.0)	
High school	38 (33.0)	
Junior college	25 (21.7)	
Bachelor degree or above	29 (25.2)	
Family income		
Low income	24 (20.9)	
Middle income	55 (47.8)	
High income	26 (22.6)	
Missing	10 (8.7)	

*Brief RCOPE, brief religious coping method; BDI, Beck Depression Inventory-II. SF-36 score = The Short Form Health Survey-36 score. Low income (< $5,000); Middle income ($5,000 to $49,999); High income (> $50,000); Missing (did not disclose income)*.

Table 2 shows that in the multivariate linear regression analysis of predictors of BDI scores, the gender of caregivers was an independent predictor of BDI scores. The positive religious coping score was an independent predictor of the BDI score (β = −5.365, *p* = 0.006). In addition, the negative religious coping score was also an independent predictor of the BDI score (β = 4.812, *p* = 0.017). Table 3 shows the analysis of the correlation between the quality-of-life score and the positive or negative religious coping score. The results suggested that the three dimensions of Vitality, Social Functioning, and Mental Health were significantly positively correlated with positive religious coping methods. The negative religious coping score was significantly negatively correlated with the Mental Health score.

**Table 2:** Multivariate linear regression analysis of predictors of depression score.

**Predictors**	**β (Regression coefficient)**	***P***
Gender (female)	3.158	0.021
Age	0.320	0.239
Positive coping method	−5.365	0.006
Negative coping method	4.812	0.017

**Table 3:** Spearman coefficients for the correlations between quality of life scores and positive and negative religious coping scores.

**Quality of life dimensions**	**Positive coping method**		**Negative coping method**	
	**R (correlation coefficient)**	**P**	**R (correlation coefficient)**	**P**
Physical function	0.007	0.938	0.050	0.597
Role-physical	−0.053	0.573	0.106	0.261
Bodily pain	−0.084	0.371	0.053	0.575
General health	−0.070	0.455	0.071	0.451
Vitality	0.228	0.014^*^	0.081	0.389
Social functioning	0.228	0.014^*^	0.002	0.985
Role-emotional	−0.111	0.236	−0.007	0.938
Mental health	0.318	0.001^*^	−0.219	0.019^*^

* *p <0.05*.

## Discussion

This study evaluated the correlation between parents' positive and negative religious coping methods and their psychological distress and quality of life, which was the first study on this aspect. Many studies have been conducted on the relationship between positive or negative religious coping methods and several health outcomes in recent years (13–15). However, the influence of the infantile patient's caregivers' religious beliefs on the caregivers was rarely noticed by clinicians or psychologists and used rationally. A survey conducted by a group of doctors in Brazil found that only 4% of them had received training in religious knowledge (16). Therefore, there is an urgent need for research on the impact of religious coping methods on caregivers of infants with CHD.

In our research, positive or negative religious coping methods were significantly correlated with depressive psychology. First, there was a negative correlation between positive religious coping method scores and the depression symptom scores, indicating that the more positive religious thoughts and religious coping methods were used, the less likely participants were to experience depression. Second, our research showed a significant positive correlation between negative religious thoughts and religious coping methods and depression scores, which indicated that as negative religious thoughts and religious coping methods increased, depression increased among the parents of infants with CHD. This study indicated a correlation between religious beliefs and depression, and this result was similar to that of Chong and his team (17). Among the parents of infants with CHD, experiencing depressive symptoms might make it more difficult for them to establish close contact with medically fragile infants, affecting the infant's care process. Therefore, the religious coping method was regarded as an intervention that could be taken. Medical assistants could use positive religious coping methods to alleviate parents' depression, such as social workers and psychologists encouraging parents of infants with CHD with a fixed religious belief to actively participate in organized or non-organized religious activities, prayer, religious reading, or gatherings of religious members.

In addition, our research showed that positive or negative religious coping methods were related to the quality of life of the parents of infants with CHD. Our research showed a significant positive correlation between positive religious coping methods and Vitality, Social Functioning, and Mental Health scores. We also found a significant negative correlation between negative religious coping methods scores and mental health scores. With the development of the social economy, people have increasingly higher requirements for quality of life. Quality of life has become an essential indicator for assessing health and living standards. Leow et al. showed that social support was positively related to the quality of life of nursing staff, and strengthening social support interventions could improve the nursing ability and quality of life of patients' family members (18). The support of religious belief was also a particular form of social support. A study in India found that if the caregiver had no religious belief or had a weak religious belief, the caregiver would show lower social function (19). The study from Hexem and his team also supported this view (20). The parents of infants with CHD in this study yielded similar results on religious coping methods and quality of life. Nevertheless, the results of this study on mental health, social functioning and positive religious coping methods were significantly positively correlated, which was different from the result of Paulo et al. (21). However, based on these similar results, it could be concluded that in the three dimensions of quality of life, positive religious beliefs could be an important intervention to improve parents' quality of life with CHD.

A proactive understanding of the religious beliefs of the parents of infants with CHD, together with the intervention of some positive religious coping methods, could help those parents who are in pain, support them spiritually, and improve their situations to some extent. In our opinion, these practices could be applied to parents of infants with other chronic diseases, and similar results might be obtained.

### Limitations

There were several limitations in this study. First, this study was a descriptive cross-sectional study. Second, the evaluation indicators of this study were primarily subjective. When filling out the questionnaire on the spot, parents were distracted by caring for their infant; they might not have been thinking deeply and might have completed the questionnaire quickly. These behaviors might bias the results. In addition, although the RCOPE has been validated in Asia and has shown good reliability and validity, the RCOPE might not accurately assess traditional religious beliefs in the East because it was developed in a predominantly Christian and American population. Finally, the parents' psychological distress and quality of life might be affected by other factors, such as the severity of the child's disease, whether there was support from a medical institution, burnout syndrome, etc. The severity of the disease might affect the mental state and be the main factor of quality of life (22). This research was not aimed at the impact of a particular religious belief but at the level of using positive religious or negative religious coping methods. Further longitudinal studies on positive or negative religious coping methods should be completed in the future to better understand the impact of religious coping methods on parents of patients with CHD and then apply religious coping, which is often ignored, to parents who have religious beliefs and are in pain.

## Conclusion

Our results support the possibility that positive or negative religious coping methods might be associated with psychological distress and quality of life in parents of infants with CHD. It was suggested that more attention should be given to the influence of religious coping methods on parents of infants with CHD, and the use of religious resources should be encouraged among those parents.

## Data Availability Statement

The original contributions presented in the study are included in the article/supplementary material, further inquiries can be directed to the corresponding author/s.

## Ethics Statement

The studies involving human participants were reviewed and approved by the Ethics Committee of Fujian Maternity and Child Health Hospital. The patients/participants provided their written informed consent to participate in this study.

## Author Contributions

J-FL and QC designed the study, performed the statistical analysis, participated in the operation, and drafted the manuscript. W-PX and W-HL collected the clinical data. HC provided financial and technical support. All authors read and approved the final manuscript.

## Conflict of Interest

The authors declare that the research was conducted in the absence of any commercial or financial relationships that could be construed as a potential conflict of interest.

## Publisher's Note

All claims expressed in this article are solely those of the authors and do not necessarily represent those of their affiliated organizations, or those of the publisher, the editors and the reviewers. Any product that may be evaluated in this article, or claim that may be made by its manufacturer, is not guaranteed or endorsed by the publisher.
